# The Effect of the FIFA 11+ Warm-Up Program on Knee Instability and Motor Performance in Male Youth Soccer Players

**DOI:** 10.3390/s25082425

**Published:** 2025-04-11

**Authors:** Badis Soussi, Tamás Horváth, Zsombor Lacza, Mira Ambrus

**Affiliations:** Research Center for Sports Physiology, Hungarian University of Sports Science, Alkotás u. 42-48, 1123 Budapest, Hungary; badis.delrio@gmail.com (B.S.); horvath2.tamas@tf.hu (T.H.); lacza.zsombor@tf.hu (Z.L.)

**Keywords:** knee, motor performance, injuries, training

## Abstract

This study aimed to investigate the effect of the FIFA 11+ program on knee instability and motor performance in male youth soccer players. Thirty male youth soccer players were divided into two groups: the experimental group (FIFA+) performed the FIFA 11+ program for 10 weeks, while the control group followed their usual warm-up routine. Dynamic knee valgus (DKV) and squat depth were assessed using a Microsoft Azure Kinect camera and dynaknee software. Maximal isometric muscle force was measured with a dynamometer. The Y Balance test was used to evaluate dynamic balance, while a countermovement jump test assessed lower limb power. The knee range of motion was measured with a goniometer, and the *t*-test was used to evaluate agility. After the intervention, the FIFA+ group showed a significant decrease in DKV and squat depth (*p* < 0.05), while the control group showed no significant changes (*p* > 0.05). Both groups improved in motor performance, with slight progress noted in the FIFA+ group. However, neither group demonstrated significant improvement in dynamic balance (*p* > 0.05). While the FIFA 11+ program may not substantially enhance overall motor performance or match the effectiveness of other training regimens, it shows potential for addressing biomechanical deficiencies and reducing the risk of injuries, particularly those related to dynamic knee valgus.

## 1. Introduction

Knee injuries such as anterior cruciate ligament (ACL) ruptures and meniscus tears are among the most common and debilitating injuries in soccer, affecting players at all levels of competition [1]. ACL injuries often occur due to non-contact situations, such as when players plant their foot and twist, land awkwardly after a jump, or suddenly decelerate during high-speed play [2]. Among children and adolescents, it is important to note that the incidence of ACL injuries has increased significantly in recent years [3]. Due to the musculoskeletal immaturity of this population, there is a pressing need for greater attention compared to adults, as injuries at such a young age can lead to unforeseen complications and may severely limit a child’s future sports career [4].

Different studies showed that knee valgus and the tibia rotation could be the main causes of ACL injury [5]. Dynamic knee valgus (DKV) is a biomechanical phenomenon strongly associated with an increased risk of ACL injuries, particularly in soccer players [6], it is characterized by the inward collapse of the knee during weight-bearing activities, often resulting from poor neuromuscular control and imbalances in strength, especially in the hip and quadriceps muscles [7]. This movement pattern places excessive strain on the ACL by increasing valgus and rotational loads on the knee joint [8]. Research shows that DKV is often associated with neuromuscular control deficits, such as weak hip abductors and external rotators, which fail to stabilize the pelvis and femur during dynamic activities [9]. Additionally, younger players undergoing growth spurts may experience altered biomechanics and motor control, further increasing the risk of valgus movement at the knee [7].

Injury prevention has become a major subject in football over the past few years [10]. Besides training and warm-up programs for preventing general injuries, specific programs for the prevention of knee injuries have been published for various types of sports such as soccer [10]. The FIFA 11 + program was developed in 2006 to address this matter, under the leadership of the FIFA Medical Assessment and Research Centre and in collaboration with the Oslo Sports Trauma Research Center and the Santa Monica Orthopaedic and Sports Medicine Center [11]; it aims to prevent injuries among soccer players. This 30 min routine is designed to be performed twice a week and requires no specialized equipment. It comprises 15 exercises divided into three components: first, running exercises; second, strength plyometric and balance exercises; and, third, running with high intensity exercises [11]. This training program has the potential to lower football player injury rates [12]. In their study, Dix et al. (2008) [13] found that a collegiate female soccer player who participated in the FIFA 11+ for 12 weeks saw improvements in their hip adduction angle and knee valgus collapse after making a 90° cut to their non-dominant leg. Additionally, Kilding et al. (2008) [14] found improvements in leg power and speed over 20 m and a tendency towards improved agility and stability in comparison to the control group after the intervention. Several authors confirmed that such improvements may contribute to the injury prevention effect of the FIFA 11+ program. In a systematic review, Sadigursky et al. (2017) [15] assessed the effectiveness of the FIFA 11+ for soccer players, revealing that the implementation of the program resulted in a 30% decrease in injuries among soccer players. Unlike other warm-up routines, FIFA 11+ comprises 15 structured exercises targeting core stabilization, eccentric thigh muscle training, proprioceptive training, dynamic stabilization, and plyometric drills, all performed with proper postural alignment; this specificity addresses the common injury mechanisms in soccer, making it more effective for players [15].

There is insufficient information which shows this program is more effective than other programs to decrease injuries for young male soccer players [12]. To the best of our knowledge, there has been no research examining the effects of the advanced FIFA 11+ program on the reduction of dynamic knee valgus (DKV), enhancement of lower limb biomechanics, and knee stability, while simultaneously improving motor performance in male youth male soccer players under 15 years old. There is a lack of comprehensive data to determine whether this program is more effective in preventing injuries and enhancing neuromuscular capabilities in male young soccer players compared to other preventive strategies. It is crucial to clarify the influence of the FIFA 11+ program on kinematic risk factors, such as dynamic knee valgus, in young male soccer players, as well as to provide conclusive findings in this domain. Furthermore, due to the serious consequences of lower extremity injuries, including long-term disability and significant financial burdens on teams, particularly for young athletes, it is essential to identify effective risk factors such as knee instability, muscle strength, balance, proprioception, and agility to mitigate injury rates and enhance performance in this demographic. Consequently, this study aims to assess the effects of the FIFA 11+ program on knee instability and motor performance among male youth soccer players. The research hypothesis posits that the FIFA 11+ program will lead to improvements in knee stability, thereby reducing dynamic knee valgus and enhancing motor skills in male young players; we anticipate that the intervention group (FIFA+) will demonstrate significant enhancement compared to the control group (regular training).

## 2. Material and Methods

### 2.1. Participants

This study recruited a convenient sample of 30 male youth soccer players from the U15 and U14 age categories (male youth players under 15 and 14 years old, age = 13.53 ± 0.51) from a local soccer team. The inclusion criteria were healthy participants aged 13 to 14 years, playing soccer three to four times per week and having a game once per week for a minimum of six months. Participants were excluded from the study if they had suffered any lower extremity and trunk injury, ACL injury or surgery within the past 6 months. Participants were randomly assigned to the Exercise Group (FIFA+) which was assigned the 10-week FIFA 11+ program with 2 sessions per week and to the control group, which used their ordinary training during the same period of intervention. Players’ physical characteristics and sport experience are presented on Table 1. Parents and the team guardian provided informed consent, which detailed the study’s purpose, protocols, and procedures. Before participation, all procedures were explained to each player, and informed consent was obtained from parents and the team guardian, then the players were randomly assigned to the control (n = 15) and FIFA+ groups (n = 15). The study protocols were approved by the Research Ethics Committee of the University of Physical Education of Budapest. (Number: TE-KEB/05/2024, date: 20 March 2024).

### 2.2. Procedures

The subjects were grouped to perform the pre-test and attended according to the chosen hours. The testing occurred in the afternoon during a week before and after the intervention, when all players were present for different measurements. The sessions began with measuring the athletes’ standing body height and body mass with a Seca altimeter 213 (Seca GmbH & Co. KG company, Hamburg, Germany) and MEDISANA PS 470 scale (MEDISANA GmbH, Carl-Schurz-Str. 2, 41460 Neuss, Germany). After that the athletes warmed up their bodies with a dynamic standard for a few minutes, we started the main measurements.

**Figure 1:**
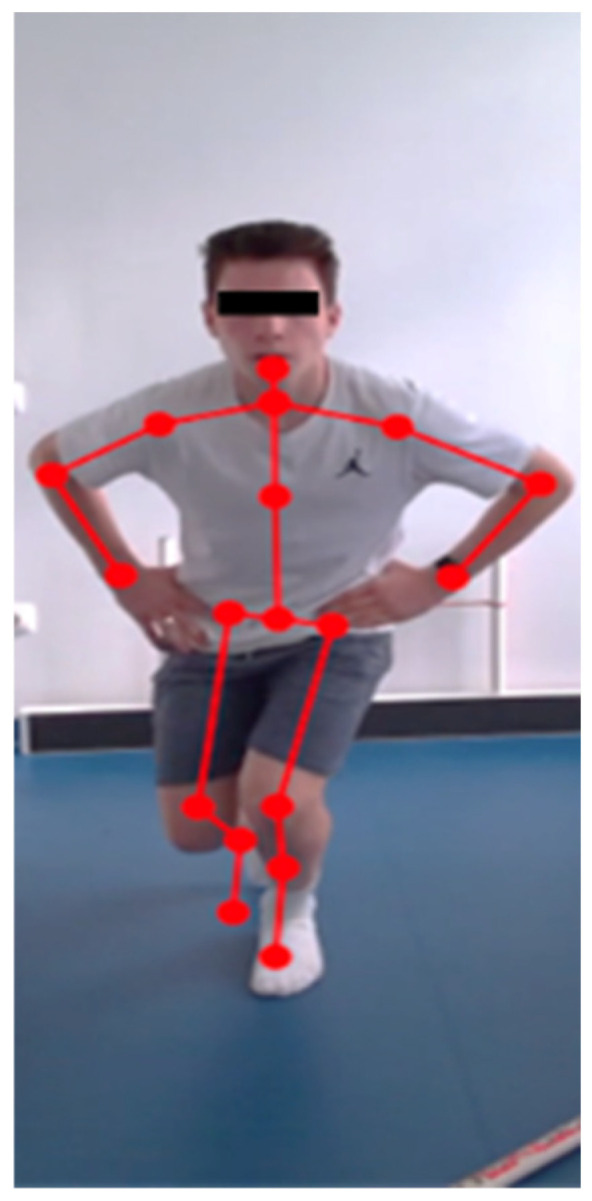
Assessment of DKV. Subject performing a single leg squat test with a markerless motion analysis system.

To evaluate kinematic parameters and measure the dynamic knee valgus, a Microsoft Azure Kinect camera (Microsoft Corp., Redmond, WA, USA) was used. The camera was positioned 250 cm away from the subjects and 100 cm above the ground, which provided optimal conditions to capture full-body images as shown in Figure 1. All data were collected using a custom software application (DynaKnee, OrthoSera Medical Zrt, Budapest, Hungary) designed for the Windows 10 operating system, which enabled data management, recording, and analysis [16]. The participants were asked to remove their shirts to allow the software to identify 18 biomarker points on their bodies. They started in a quiet standing position with their hands on their hips to obtain baseline data for the length of their lower extremities. For the movement excursion, the subjects bent their left knee to a 90-degree angle, lifting their left lower leg parallel to the ground. To measure the DKV shift, ten technically correct single-leg squats on each side were recorded and the angles of the dynamic knee valgus were quantified at 15% of the maximum squat depth, participants had to execute squats as low as possible while keeping their heels and feet in contact with the ground. This heavy knee load provoked a DKV under tension. Squats were considered valid if the participants maintained their balance throughout the repetition with their hands on their hips and did not step away during the examination. If a trial was improperly executed, the subjects were asked to repeat it.

For the maximum isometric muscle force measurements, the Hoggan MicroFET3 wireless dynamometer (Hoggan Scientific LLC, Salt Lake City, UT 84104, USA) was used; the targeting muscles were the hamstring and quadriceps. The subject was instructed in the movement and the maximum force was measured in three attempts after a verbal start signal. First, knee extension was measured, where the subject was placed in a seated position with the back of the knee against the edge of the bench. The leg was hanging down, the device was placed on the chin and the hands were placed on the thighs to avoid additional use of other muscles. The subject was then directed to gradually apply maximum force by extending the knee against an unyielding resistance, during which the dynamometer measured the peak force output. Next, knee flexion was measured, where the subject lay in the prone position with one leg extended. The other leg to be measured was bent at a 90-degree angle. Arms were bent and placed behind the back and the subject was instructed to not lift their hips off the bench; the participant may be positioned either lying face down or seated, with the knee flexed at a 90-degree angle. The dynamometer is positioned above the ankle on the back side of the leg, and the participant is instructed to exert maximal force by flexing the knee against the applied resistance.

The lower limb power was assessed by a gold standard opto-electric cells system (OptoJump, Microgate, Bolzano, Italy). In the present study, lower limb power was analyzed by performing the counter movement jump test. The starting position was standing with hands on hips. The participants executed three consecutive explosive vertical jumps with minimal ground contact time to enhance lower-body power and reactive strength. The exercise commenced from an upright position, with the feet positioned at hip-to-shoulder width. This was followed by a swift downward countermovement, during which the knees flexed to approximately 90 degrees and the hips descended, enabling the muscles to accumulate elastic energy. Without any pause, the participant extended the hip, knees, and ankles in a coordinated triple extension to launch upward, ensuring a straight body alignment at the moment of takeoff. Upon landing, the player absorbed the impact gently on the feet, allowing for a slight knee bend before promptly initiating the subsequent jump, utilizing the elastic energy stored during the stretch-shortening cycle. This process was repeated for the second and third jumps, highlighting the importance of rapid transitions, minimal ground contact time, and continuous movement. Throughout the entire sequence, the core remained engaged, the chest was kept upright, and the arms aided in generating momentum, thereby maximizing jump height and facilitating efficient power transfer. The final landing was executed with control, involving flexion of the knees and hips to safely absorb the impact, thereby minimizing undue stress on the joints and reinforcing proper landing techniques.

This study employed the Y-Balance Test (YBT) to evaluate dynamic balance. The YBT Kit was used to conduct the assessments. Participants were instructed to maintain a one-legged standing position, with their hands resting on the hips of the leg that was on the platform, ensuring that the big toe touched a designated horizontal line. Using the free limb, they were required to move a block in three specified directions: anterior (ANT), posterolateral (PL), and posteromedial (PM), aiming to reach the furthest point possible before returning to the initial position. Participants alternated limbs for each direction while maintaining the starting position of the loaded limb, with permitted movements of the foot, such as separation of the heel or forefoot. Any body movement was allowed, and adjustments were made to normalize for lower limb length and ensure consistent platform height. The length of the lower limbs was measured while the subjects were positioned and ensuring the legs were straight to align the pelvis. Measurements were taken from the upper anterior iliac spine to the medial malleolus of the lower limb. Each subject completed three repetitions for both the left and right lower limbs across all directions. A trial was deemed unsuccessful if the participant lost balance, failed to return to the starting position, stepped off the platform, or lost contact in an uncontrolled manner, particularly if the loaded foot moved beyond the horizontal line. The distance the platform was displaced was recorded as the result for the respective limb in a specific direction. The reach distance was measured as the distance from the contact point to the center, in centimeters. The normalized average of the three repetitions was recorded as the final score.

Angle of knee range of motion was measured in a supine position by a goniometer. The hip and knee of the dominant limb flexed at 90° (initial position: 0°), with the foot relaxed and the contralateral leg extended. The participant is positioned supine on a flat surface with their legs extended. The examiner positions the goniometer’s axis (fulcrum) at the lateral epicondyle of the femur, ensuring that the stationary arm is parallel to the lateral midline of the femur and directed toward the greater trochanter of the hip. Meanwhile, the movable arm is aligned with the lateral midline of the fibula, pointing toward the lateral malleolus (ankle bone). The participant is then asked to actively flex the knee by bringing the heel toward the buttocks as far as possible. The examiner gently assists in moving the leg into maximum knee flexion. The angle formed between the femur and tibia is then recorded. Proper stabilization of the hip and pelvis is crucial to prevent compensatory movements that may compromise the accuracy of the measurement.

**Figure 2:**
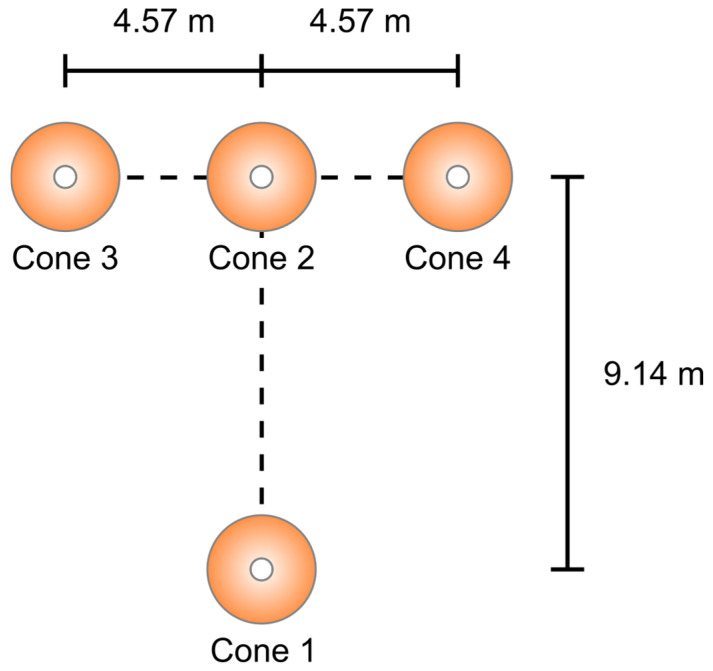
Cone placement to guide the agility *t*-test.

In our study, agility was assessed by *t*-test. The assessment is carried out on a non-slip surface and features a T-shaped running course delineated by four cones. The configuration includes a starting cone (1) located at the base of the “T”, as shown in Figure 2, with three additional cones that form the top: a central cone (2) situated 9.14 m directly ahead, and two lateral cones (3 and 4) placed 4.57 m to the left and right of cone 2, respectively. The participant initiates the test at cone 1, adopting a two-point starting position. Upon receiving the tester’s signal, the individual sprints 9.14 m to cone 2. After reaching cone 2, the player executes a rapid lateral shuffle to the left towards cone 3, touches it with their left hand, then shuffles laterally to the right to cone 4, making contact with it using their right hand. Subsequently, the participant shuffles back to cone 2, touches it once more, and then backpedals to the starting cone (1) to conclude the test. The total duration from commencement to completion is recorded using a stopwatch, with shorter times reflecting superior agility performance. Failure in touching the base of the cone or in facing forward all through the test and crossing over of one leg in front of the other leg leads to rejection of the trial. The best value, to the nearest 0.1 s, of the three successful trials is taken.

### 2.3. Intervention

The intervention group (FIFA+) performed the FIFA+ injuries prevention warms up for 10 weeks with 2 sessions per week (Monday and Friday each week); it is a comprehensive warm-up program developed by the FIFA Medical Assessment and Research Centre and designed to reduce injuries in soccer players, particularly non-contact injuries like ligament tears and muscle strains. The program emphasizes neuromuscular control, strength, balance, agility, and proper movement patterns; it is divided into three parts.

The first part (6–8-min), running drills performed over a 20 m course, focuses on dynamic warm-up, it includes: (1) straight running, jog forward at a moderate pace over 20 m, then return (2 sets); (2) running with hip out, jog forward, bringing the knee up and rotating outward to open the hips (2 sets); (3) running with hip in, jog forward, lifting the knee and rotating inward (2 sets); (4) circling partner, jog in pairs, stopping every few meters to circle around each other before continuing (2 sets); (5) shoulder contact, jog side by side with a partner, lightly bumping shoulders at designated points (2 sets); (6) quick forward and backward runs, sprint 5 m forward, then jog 3 m backward before repeating (2 sets).

The second part (10–12 min), strength, balance, and plyometrics, targets strength, balance, and dynamic stability, it includes: (1) the bench (plank), hold a plank position for 30 s. More advanced levels involve lifting one arm or leg (2 sets of 30 s). (2) Sideways bench (side plank), hold a side plank for 30 s on each side. Advanced versions include adding hip dips or leg lifts (2 sets of 30 s per side). (3) Hamstring strengthening (Nordic hamstring curl), slowly lower the upper body while keeping the knees on the ground, resisting the fall. Players progress by reducing hand support (3 to 5 repetitions). (4) Single-leg strength (squats), perform controlled squats on one leg (2 sets of 30 s). (5) Jumping and landing control, perform two-legged vertical jumps, focusing on a soft landing with progress to lateral jumps and single-leg jumps (2 sets of 30 s). (6) Bounding (high jumps), jump forward with controlled landings and progression involves single-leg bounding and maximal jump height (2 sets of 30 s).

The third part (6–8 min), running, includes: (1) rapid change of direction with sprint 10 m forward, quickly cut to the left, sprint another 10 m, and then cut to the right before stopping (2 sets); (2) bounding and cutting with sprint forward while incorporating bounding steps, then making a sharp cut to the side (2 sets); (3) plant and cut with sprint forward, plant one foot firmly, then cut 90 degrees to the side before sprinting again (2 sets).

The control group was instructed to adhere to their standard warm-up routine, performing it without any limitations, the training included a general warm-up with light jogging, dynamic stretching, and strength and conditioning exercises such as squats, lunges, and general resistance exercises.

### 2.4. Statistical Analysis

The data in this study are presented as mean ± standard deviation (SD). Normality of the pre- and post-data was checked using the Shapiro–Wilk’s normality test. To analyze the differences within groups, paired-sample *t*-test and the non-parametric Wilcoxon signed rank test were employed depending on data normality. Significance level for all statistical tests was set at *p* < 0.05. Data analysis was performed using JASP version 0.16.4.0 (JASP, Department of Psychological Methods, University of Amsterdam, Amsterdam, The Netherlands).

## 3. Results

**Figure 3:**
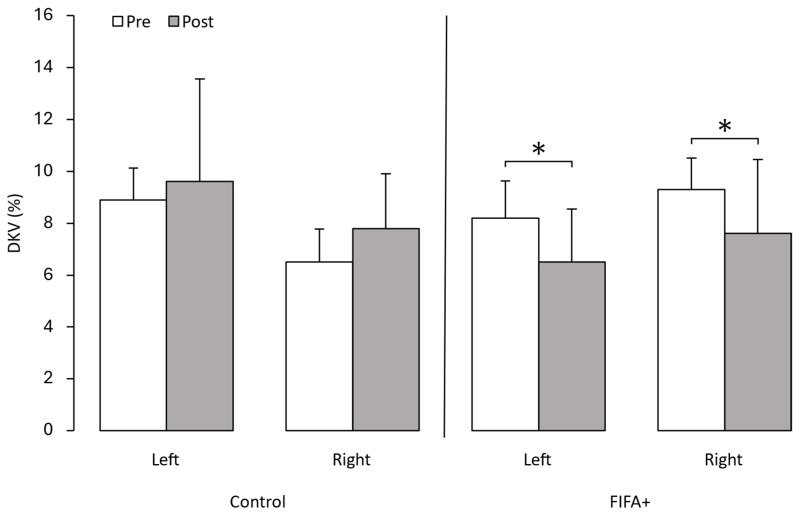
Dynamic knee valgus angle before (Pre) and after (Post) intervention period of 10 weeks. Bars represent mean values; error bars are standard errors. Asterisks denote significant difference between Pre and Post values. Dynamic knee valgus is measured in percentage (%) to normalize values relative to individual body dimensions.

**Figure 4:**
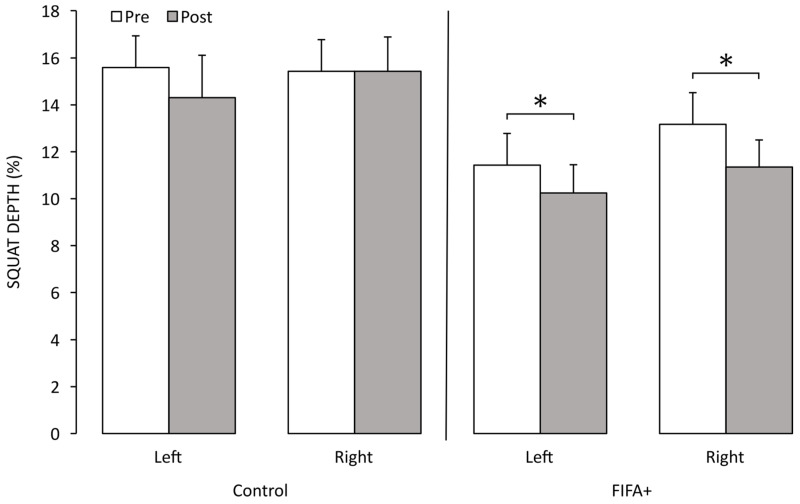
Squat depth before (Pre) and after (Post) intervention period of 10 weeks. Asterisks denote significant difference between Pre and Post values. Bars represent mean values; error bars are standard errors. Squat depth is measured in percentage (%) to normalize values relative to individual body dimensions.

The FIFA+ group demonstrated a significant decrease in DKV on both sides (*p* < 0.05) (Figure 3), same as for squat depth (*p* < 0.05) (Figure 4), and this finding concluded that the intervention was effective to decrease the DKV and improve knee stability, with a greater squat depth being recorded. On the other hand, the control group showed an increase in DKV, which is a risky sign.

Table 2 displays the results of the paired-sample *t*-test within each group for the maximum isometric strength during flexion and extension (hamstring and quadriceps) for each group. The FIFA+ group showed a significant improvement in both sides for flexion (*p* < 0.05), and extension (*p* < 0.05) after 10 weeks of training. For the control group, an improvement was recorded for flexion (*p* < 0.05) and extension (*p* < 0.05) only for the dominant leg (right). The table also indicates no significant differences on hamstring/quadriceps ratio (H/Q) in both legs for both groups, the ratio remained stabilized with a very slight change.

Finally, Table 3 displays the results of the paired-sample *t*-test and the Wilcoxon signed rank test, it demonstrates no significant differences in the pre- and post-test measurements of Y balance scores for both groups (*p* > 0.05); however, the FIFA 11+ group showed an improvement in lower limb power (*p* < 0.05), knee range of motion (*p* < 0.05), and agility (*p* < 0.05), while no significant change was recorded for the control group (*p* > 0.05).

## 4. Discussion

This research aimed to examine the impact of a 10-week training of the FIFA 11+ program on dynamic knee valgus and motor performance among youth soccer players. The hypothesis was that the FIFA 11+ program would yield positive outcomes for the FIFA+ group when comparing pre- and post-test results, in contrast to the control group, which engaged in a standard warm-up routine. The key findings indicate that following the 10 weeks of intervention, the FIFA+ group recorded an improvement in dynamic knee valgus (DKV) and squat depth, meanwhile the control group did not record any improvement, only increased DKV values. Additional results revealed that the FIFA 11+ program led to improvements in the maximum isometric force of hamstring and quadriceps muscles, same for lower limb power, knee range of motion, and agility; however, for the control group, only an improvement in maximum isometric force of hamstring and quadriceps muscles of the right leg was recorded. Furthermore, there was no enhancement in dynamic balance for either group.

Related to dynamic knee valgus and squat depth, the findings align with numerous studies. For example, Seyedi et al. (2023) [12] explored the influence of an eight-week FIFA 11+ program on the knee kinematics and proprioception of both male and female soccer athletes. Their results suggested that the FIFA 11+ program possesses significant potential to enhance knee valgus in adolescent soccer players while mitigating neuromuscular and certain biomechanical risk factors associated with sport-specific activities. Moreover, Steffen et al. (2013) [17] examined the effects of the FIFA 11+ program on female players over a season, revealing that exercises aimed at improving core stability and proprioceptive training within the FIFA 11+ framework effectively target modifiable risk factors such as DKV. The alignment observed between the training research program and the current research may be attributed to their shared focus on neuromuscular control, which plays a vital role in stabilizing the knee joint through the modulation of muscle responses informed by mechanical sensors [18]. Several exercises in the FIFA 11+ program support the neuromuscular control of the lower extremities, particularly targeting the hip abductors (gluteus medius and minimus); these muscles play a crucial role in stabilizing the pelvis and maintaining proper knee alignment during dynamic movements [19,20]. Furthermore, several exercises (specifically exercises 5, 6, 9, and 11–14) within the FIFA 11+ program are categorized as resistance training aimed at enhancing the strength of lower extremity muscles; consequently, it is plausible that the observed improvements in knee valgus among the participants in the current study can be attributed to the increased power and strength of the lower limbs [21,22].

Regarding the squat depth, our finding was in line with numerous studies which have suggested that neuromuscular training programs can effectively enhance squat depth while simultaneously mitigating knee valgus by targeting fundamental strength and mobility deficiencies [12,17]. Moreover, the development of the glutes, quadriceps, hamstrings, and core stabilizers is essential for preserving correct squat mechanics, thus enabling individuals to achieve greater depth while maintaining stability [23].

Our results diverged from certain previous studies. Kilding et al. (2008) [14] indicated that after a six-week training program, there were no significant alterations in valgus alignment for the intervention group (FIFA+ group), suggesting that extended durations may be required. Additionally, Soligard et al. (2010) [24] observed that while the FIFA+ program led to an overall reduction in injury rates, specific biomechanical enhancements, such as dynamic knee valgus, were less evident in groups with low adherence. These findings may imply that the program’s lack of specificity regarding knee injuries and its duration could be contributing factors to the absence of significant improvements, which contrasts with our findings.

Related to motor performance in the current study, the FIFA 11+ program significantly improves the strength of the hamstrings and quadriceps muscles, resulting in enhanced functional performance and a more advantageous hamstring-to-quadriceps (H/Q) ratio. Our findings align with previous research, including a study by van der Horst et al. (2015) [25], which demonstrated a notable increase in eccentric hamstring strength among soccer players who followed the FIFA 11+ protocol over a 10-week period. Additionally, research conducted by Daneshjoo et al. (2013) [26] revealed substantial improvements in both concentric and eccentric quadriceps strength after 8 weeks of the FIFA 11+ program in young soccer players. The FIFA 11+ program incorporates various plyometric and strength exercises, such as Nordic hamstring curls, single-leg strength drills, lunges, and squats, which are specifically designed to target and enhance the strength of the hamstrings and quadriceps [17,27].

Improvements in lower limb power were observed in this study, corroborating the findings of Daneshjoo et al. (2012) [26], who reported that male soccer players exhibited significant enhancements in countermovement jump height and explosive lower limb strength following eight weeks of FIFA 11+ training. Additionally, Gatterer et al. (2012) [28] highlighted that players were able to sustain their improvements in jump and sprint performance after integrating the FIFA 11+ program into their seasonal training regimen. The FIFA 11+ program incorporates plyometric exercises which specifically enhance lower limb power. Furthermore, agility drills within the FIFA 11+ program contribute to the development of explosive lower limb power while simultaneously enhancing neuromuscular coordination [17].

The intervention program demonstrated effectiveness in enhancing the range of motion, corroborating findings from prior research. Hübscher et al. (2010) [29] conducted a systematic review and meta-analysis of injury prevention programs, revealing that initiatives such as FIFA 11+ and FIFA+ Kids can significantly enhance joint flexibility, particularly in the knee and hip areas. Furthermore, Myer et al. (2011) [30] indicated that injury prevention programs focusing on strength and flexibility, such as the FIFA 11+ program, contribute to improved knee function by increasing range of motion.

In the current study, agility was assessed as part of a functional evaluation, revealing a notable enhancement in the FIFA+ group following the intervention. This outcome aligns with various studies. McCall et al. (2015) [31] explored the effects of injury prevention programs, including FIFA 11+, on the physical performance of soccer players, reporting significant improvements in agility after the implementation of the warm-up regimen. Similarly, Della Villa et al. (2020) [32] found that FIFA 11+ contributed to the enhancement of lower-limb mechanics, including agility, among soccer players. The components of FIFA+, particularly the third part focused on change of direction drills, are effective in enhancing agility. In addition, the synergy of increased strength and joint stability in prevention programs facilitates athletes in executing quick, controlled movements with greater efficiency and speed [33].

This investigation did not reveal any enhancements in dynamic balance for either group, and our results diverged from those of prior research, which indicated that improvements in balance are a significant outcome of the FIFA 11+ program. Regular engagement with this program was found to be effective not only in decreasing injury rates but also in improving balance and neuromuscular function [34,35]. Conversely, our findings align with certain studies [36,37], which suggested potential reasons for the lack of improvement in dynamic balance, although the FIFA 11+ program incorporates balance exercises, these may not be sufficiently targeted or demanding to elicit significant enhancements. Dynamic balance is heavily dependent on the body’s capacity to maintain stability during movement, which often requires more intricate proprioceptive and coordination skills than those typically involved in standard warm-up routines [38,39]. Overall, the relationship between dynamic balance and the reduction of knee valgus has produced inconsistent findings [40].

The FIFA 11+ program has shown efficacy in improving motor performance; however, it may not possess the level of specialization that certain programs designed for elite athletes or specific motor performance goals offer. This insight elucidates the absence of progress observed in dynamic balance and the overall improvements seen in the control group which maintained their ordinary training. Nonetheless, regarding injury prevention, the FIFA+ program effectively addressed the biomechanical deficiencies associated with knee valgus and enhanced knee stability.

## 5. Limitations

There are some limitations in the current study. First, not considering players’ position and status (starting or reserve players); second, using the stopwatch to measure timing might provide some errors despite this method having an acceptable relative reliability; and, third, it would be necessary to add more weeks for intervention which might be beneficial to record improvements on dynamic balance.

## 6. Conclusions

The FIFA 11+ program has demonstrated its effectiveness as an intervention aimed at improving dynamic knee valgus (DKV) and enhancing motor performance among young soccer players. By integrating structured neuromuscular training, balance activities, plyometric exercises, and strength training, the program significantly mitigates excessive knee valgus angles, which are often linked to a heightened risk of injuries, particularly those affecting the anterior cruciate ligament (ACL). Consistent application of the FIFA 11+ program fosters improved lower-limb alignment, better neuromuscular coordination, and increased movement efficiency, all of which contribute to enhanced stability and resilience against injuries during high-intensity soccer activities. Furthermore, the program bolsters essential motor performance characteristics, including agility, sprinting speed, jumping capability, and muscular strength, which are vital for achieving peak performance in soccer players, promoting both performance enhancement and long-term musculoskeletal health. Future research should try to compare the effectiveness of the standard FIFA 11+ program versus individualized modifications tailored to players’ specific biomechanics, skill levels, and injury history.

## 7. Implications and Future Research

We suggest that clubs should educate coaches, players, and medical staff on the long-term benefits of the FIFA 11+ program to encourage consistent use and consider it a fundamental part of performance training, not just a warm-up. Additionally, physical trainers should focus on proper technique, especially in landing mechanics, to improve players’ knee alignment and stability and we suggest that female players should also be encouraged to adopt FIFA 11+ from a young age to improve neuromuscular control and lower injury risk.

## Figures and Tables

**Table 1:** Physical characteristics and sport experience of the participants. BMI: body mass index.

	Group	Mean ± SD	*p*-Value
Body mass (kg)	control	55.1 ± 9.4	>0.05
	FIFA+	59.0 ± 6.4	
Body height (cm)	control	166.5 ± 8.9	>0.05
	FIFA+	170.4 ± 6.6	
BMI (kg/m^2^)	control	19.2 ± 1.9	>0.05
	FIFA+	20.3 ± 2.4	
Sport experience (years)	control	2.0 ± 0.4	>0.05
	FIFA+	2.2 ± 0.4	

**Table 2:** Mean standard deviation (SD) of maximum isometric force (hamstring, quadriceps).

	Left			Right		
	PRE	POST	*p*-Value	PRE	POST	*p*-Value
Extension						
G1—intervention	215.8 ± 43.3	234.1 ± 45.1	**<0.05**	217 ± 26.8	239.6 ± 27.41	**<0.05**
G2—control	190.2 ± 12.5	192.8 ± 11.1	0.15	220 ± 37.7	225.3 ± 40	**<0.05**
Flexion						
G1—intervention	174.2 ± 37.6	191 ± 34.1	**<0.05**	179.4 ± 37.8	194.9 ± 36.9	**<0.05**
G2—control	191.6 ± 47.7	191.1 ± 41.9	0.08	188 ± 33.7	191.6 ± 32.8	**<0.05**
H/Q ratio						
G1—intervention	0.84 ± 0.2	0.84 ± 0.17	0.85	0.83 ± 0.15	0.82 ± 0.18	0.38
G2—control	1.02 ± 0.28	0.99 ± 0.23	0.2	0.89 ± 0.27	0.88 ± 0.26	0.71

**Table 3:** Mean standard deviation (SD) of Y balance, knee ROM, power (range of motion), and agility.

	PRE	POST	*p*-Value
Y Balance			
Anterior			
Control	3.93 ± 2.94	3.07 ± 2.38.	0.35
FIFA+	5.2 ± 4.24	6.13 ± 4.99	0.62
Posteromedial			
Control	5.28 ± 4.51	5 ± 5.57	0.86
FIFA+	3.6 ± 5.6	8.27± 9.35	0.13
Posterolateral			
Control	7.07 ± 5.5	4.93 ± 4.07	0.22
FIFA+	8.6 ± 7.15	6.07 ± 4.08	0.28
Lower limb Power (W/kg)			
Control	13.61 ± 1.37	13.09 ± 3.69	0.85
FIFA+	13.58 ± 1.13	14.81 ± 1.13	**<0.05**
Knee ROM (degrees)			
Control	136.1 ± 6.64	136.2 ± 5.24	0.9
FIFA+	131.7 ± 5.7	135 ± 3.77	**0.02**
Agility (s)			
Control	15.06 ± 1.03	14.93 ± 1.04	0.06
FIFA+	14.96 ± 0.97	14.38 ± 1.18	**<0.05**

Value indicated using Wilcoxon signed rank test and value indicated using paired *t*-test.

## Data Availability

The data that support the findings of this study are available from the corresponding author upon reasonable request. The data obtained in this study are available on reasonable request due to privacy and ethical restrictions; once requested, the corresponding author will provide it.

## Abbreviations

DKV	Dynamic knee valgus
ACL	Anterior cruciate ligament
YBT	Y balance test
